# Evaluation of industrial water use efficiency considering pollutant discharge in China

**DOI:** 10.1371/journal.pone.0221363

**Published:** 2019-08-27

**Authors:** Rongrong Xu, Yongxiang Wu, Gaoxu Wang, Xuan Zhang, Wei Wu, Zan Xu

**Affiliations:** 1 Hydrology and Water Resources Department, Nanjing Hydraulic Research Institute, Nanjing, China; 2 State Key Laboratory of Hydrology-Water Resources and Hydraulic Engineer, Nanjing, China; 3 Hydrology and Water Resources College, Hohai University, Nanjing, China; Institute for Advanced Sustainability Studies, GERMANY

## Abstract

China is facing severe pressure on its water resources and water environments. Calculating the industrial water efficiency of each province is an important index for the central government to evaluate local governments. In the traditional water resources evaluation index, the industrial water use efficiency and pollutant discharge are evaluated separately. In this paper, we collected industrial input data, output data and pollutant discharge data with a four-stage data envelopment analysis to calculate China's industrial water use efficiency with and without considering pollutant discharge, and then analyzed the factors influencing the industrial water use efficiency. The results show that the eastern coastal provinces of China have the highest water use efficiency and are less affected by pollutant discharge than other provinces. The industrial water use efficiency of the central and western provinces is lower than that of the other provinces, and the industrial water use efficiency in the central provinces is greatly affected by pollutant discharge. Factor endowment, economic development level, scientific and technological progress, industrial structure, proportion of foreign investment, water consumption per 10000 yuan of value-added by industry, industrial sewage treatment capacity and educational investment have a significant influence on the industrial water use efficiency of China. We suggest that the government strengthen the construction of sewage plants and other related infrastructure in central provinces when conducting the industrial transfer of heavy polluting enterprises.

## 1 Introduction

China's rapid growth in industrial output has put substantial pressure on water resources and water environment[1,2]. There are significant differences in China's industrial structure, water resource factor endowment[3], and water-saving policies among provinces[4,5]. China's water consumption is mainly divided into domestic, industrial, agricultural and ecological water consumption[6]. China used 604.02 billion cubic meters of water in 2016, including 130.8 billion cubic meters for industrial use [7]. The industrial water consumption varies greatly in different regions of China. Industrial water use is the highest in eastern coastal areas, while that in the northwest is only one-tenth of that in eastern coastal cities. However, China's highest water consumption per ten thousand yuan of added value is in the middle reaches of the Yangtze river. The industrial water use efficiency in the middle reaches of the Yangtze river is low because of the weak water-saving technology and high water resource factor endowment. China discharged 73.53 billion tons of wastewater in 2015, including 19.95 billion tons of industrial wastewater[8]. The amount of industrial waste water discharged still varies widely in different parts of China. The industrial wastewater discharge in the middle reaches of the Yellow River is 10 times that in the northwest. Sewage discharge of ten thousand yuan industrial added value is also the highest in the middle reaches of the Yellow River. China's eastern coastal areas have relatively high levels of sewage discharge, but the sewage discharge of ten thousand yuan industrial added value is the lowest in China. Most of the harmful substances in industrial wastewater are discharged into water bodies, resulting in different degrees of pollution in the drinking water sources of many cities[9]. The water shortage caused by water pollution is close to 62% of the total water resources in Chongqing and Yunnan[10]. The water resource demand of industrial enterprises is increasing, and the water pollution situation is becoming more dangerous[11]. The sustainable development of industry will be limited by the current industrial water shortage and water pollution[12,13]. Some industrial enterprises have caused serious water pollution and exacerbate China's water problems. Strengthening water efficiency management in industry can not only improve the water environment but also alleviate the conflict between the supply and demand of water resources[14].

The commonly used indicators of industrial water use efficiency include water consumption of ten thousand yuan of industrial added value [15], the reuse rate of industrial water [16], and thewater quota for industrial products [17]. These indicators represent the economic benefits of water resources, but the connotations of these indicators are difficult to expand, and the indicators are unable to reflect the ecological and environmental benefits of water resources[18]. From an evaluative perspective, industrial water use efficiency is the ratio of output to input, and pollutant discharge can also be considered one kind of input. The efficiency in this paper refers to the ability to achieve the maximum output under a given input or the ability to minimize the input under a given output. This scenario is considered an example of Pareto optimality[19]. In this study, the ratio of the lowest feasible water consumption to the actual water consumption can be used for the industrial water use efficiency[20,21].

Data envelopment analysis (DEA) uses the method of linear programming to measure efficiency[22]. DEA is a nonparametric method in which the specific form of the production function does not need to be defined, and the values of the input and output are required. DEA can maximize the efficiency of the service unit. In the calculation, the unit with an efficiency value equal to one is the relatively effective unit, and the unit with a value less than one is the invalid unit[23,24]. Charnes first proposed DEA in 1978[25]. To solve the limitation of the possible convexity of the production set in the DEA model, Charnes proposed another DEA model in 1985 to evaluate the finiteness of production technology. Afterwards, with the continuous extension of the DEA model, Anderson targeted the shortcomings of the previous DEA model, and put forward the super efficiency DEA model (SE-DEA)[26]. This model can distinguish the effective decision-making unit, and further sort the effective decision units with values greater than one.

In recent years, many scholars have conducted substantial research on evaluating water use efficiency using DEA. The traditional two-stage DEA method can evaluate the efficiency of a system[27]. Güngör-Demirci used the two-stage DEA to evaluate California's water supply system[28], and Chongfeng Ren divided 12 cities in Gansu Province into multiple water use subsystems, and analyzed the water use efficiency of each subsystem with two-stage DEA model[29]. Some scholars have improved the traditional DEA model. Gidion used network DEA to analyze the urban water use efficiency[30], Prakashan Chellattan Veettil used a stochastic DEA to analyze the efficiency of irrigation water use in the agricultural production system in the Krishna River Basin[31], Zhineng Hu combined bilevel programming with DEA to analyze the water use efficiency of ten cities in the Minjiang River Basin[32]. Bian Yiwen divided urban water use into a water system and sewage discharge system for analysis and defined urban water use efficiency as the average of the water use efficiency of the two systems[33]. Some other scholars used the DEA model to calculate China's water use efficiency when considering wastewater discharge. Shuqiao Wang used Seiford's linear converting method to calculate the water use efficiency of China and used the DEA-Tobit model to analyze the indicators affecting the water use efficiency of China[34]. Deng Guangyao used the SBM-DEA model to analyze the water use efficiency of 31 provinces in China[35].

In current research and engineering practices, the following issues exist: (1) the index of industrial water use efficiency is usually expressed as the water consumption of ten thousand yuan of industrial added value. Although this index can reflect industrial water use efficiency, it lacks expansibility and cannot reflect the impact of pollution on industrial water use efficiency; (2) Although some studies have considered the effect of wastewater discharge on industrial water use efficiency, the effect of pollutants in wastewater on industrial water use efficiency has not yet been considered. (3) The traditional DEA method fails to consider the influence of different external environments between provinces on water use efficiency, and the method needs improvement.

In this paper, technical efficiency was selected as the index of China's industrial water use efficiency. At the same time, we further improved the DEA model so that different decision-making units could be calculated in the same external environment. We calculate the industrial water use efficiency of 31 Chinese provinces with and without considering pollutant discharge, and analyzed the factors affecting the industrial water use efficiency.

## 2 Materials and methods

### 2.1 Industrial water use efficiency and the principle of data envelopment analysis

Industrial water use efficiency belongs to the category of efficiency, which is the ratio relation between input and output. In this paper, industrial water use efficiency is defined as the ratio between the actual industrial output and the ideal optimal output under the selected input of each decision-making unit, the efficiency value is between 0 and 1. Closely related to industrial water use efficiency is the production of industrial wastewater which contains pollutants that will cause damage to the water environment. However, reducing emissions requires more investment (such as upgrading production technology or installing sewage treatment equipment) or output control (such as banning high-pollution projects or shutting down high-pollution factories). Provinces that do well in environmental protection allocate inputs that can be used for production that includes pollution control activities, this will increase input or decrease output. If the decline in the province's sewage is not considered, the calculation of industrial water use efficiency in the province will be biased.

Economists regard environmental capacity as a resource with the universality and non-exclusivity of supply. This is a typical feature of public goods, and pollution discharge has a strong negative externality. Provinces occupy more resources in an environmental capacity as the level of pollution increases. Some scholars evaluate methods that cause the least harmful discharge to maximize economic benefits. Therefore, industrial pollutants are applied as a factor input to study the deviation between industrial water use efficiency considering pollutant discharge and industrial water use efficiency not considering pollutant discharge.

DEA is often used in the field of economic management to analyze the relative efficiency of each decision-making unit. Its most significant advantages are that it does not need to consider the functional relationship between input and output or estimate parameters in advance, and it has strong objectivity. The envelope surface is formed by the actual input and output data, and the current production frontier is calculated. The decision-making unit at the production frontier is a Pareto optimum. Based on actual research needs, the efficiency of each decision-making unit can be calculated by changing the input and output factors of the model.

**Fig 1:**
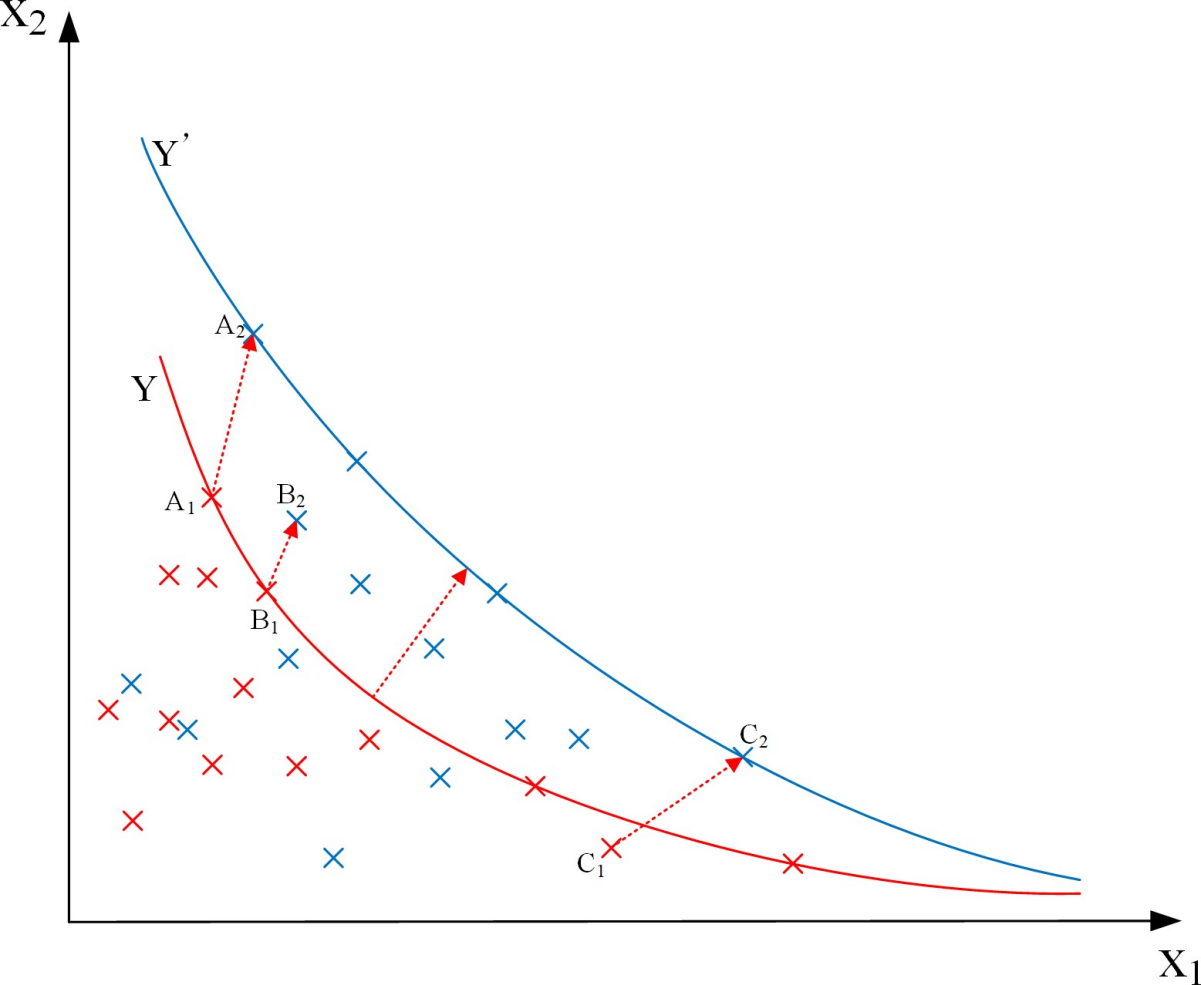
Schematic diagram of data envelopment analysis.

To explain the calculation principle of DEA, we assumed the total industrial production as the output factor (Y), industrial water consumption is the input factor (X_1_) and the other input factors are designated as X_2_. As shown in Fig 1, the red line Y and the red points are the production frontier and the actual input-output positions, respectively, formed by the current decision-making units in which pollutant discharge is not considered. A point on the red line indicates that the decision-making unit reaches the Pareto optimum, that is, the efficiency value is 1. The points not on the red line indicate that the decision-making unit did not reach the Pareto optimum and the efficiency value is less than 1. When pollutant discharge is considered, the input factor X_2_ changes, the production frontier moves from Y (red line) to Y’(blue line), and the positions of the points are shifted. We selected three possible situations in this process—decision-making units A, B and C. The decision-making unit A is transferred from A_1_ to A_2_, and both of them are at the production frontier, this indicates that the decision-making unit reaches the Pareto optimum in both situations. The decision-making unit B is transferred from B_1_ to B_2_, indicating that the decision-making unit B is in the Pareto optimal state when pollution discharge is not considered. However, when considering the emission of pollution, Pareto optimization is not reached due to the relatively large increase of pollutant input. The decision-making unit C is transferred from C_1_ to C_2_, indicating that when pollutant discharge is not considered, decision-making unit C does not reach the Pareto optimum. However, decision-making unit C has a low pollutant emission and reaches the Pareto optimal state when considering pollutant discharge. Detailed quantitative calculation processes are discussed in Sections 2.2 and 2.3

In some provinces, the efficiency ratio of input and output decreases relatively due to the importance of environmental protection. If the central government does not consider environmental factors when assessing the water efficiency of local governments, that will certainly set the wrong example. Therefore, we select appropriate indicators to further explain and improve the DEA method.

### 2.2 Indicators and data sources

(1) Input-output indicator selection

Labor and capital are indispensable in production, and industrial water consumption, as a basic input factor in the industrial production process, is the essential element of the research framework of this paper. We selected the total number of industrial employees and industrial total assets as input indicators to study industrial water efficiency.

The industrial output value can reflect the output benefits of industrial water resources, and we used the industrial sales value to represent the total industrial output value in China during 2007–2016.

(2) Pollutant indicator selection

We selected the most common pollutant indicators—chemical oxygen demand (COD) and ammonia nitrogen (NH_3_) levels. The COD and NH_3_ levels for discharge from industrial wastewater were used as pollutant indicators to evaluate the industrial water use efficiency in 31 provinces in China.

The more pollutants emitted, the more costs must be paid. In this paper, pollutant discharge is also an input factor. We compared the differences in industrial water use efficiency with and without pollution indicators.

(3) External variable indicator selection

External variables refer to factors that can affect industrial water use efficiency but are difficult to control by industry itself. Many external variables affect industrial water use efficiency, including not only macroeconomic policy factors and market regulation but also factors that can hardly be controlled in the future, such as water-related technologies.

The external variables selected in this paper were used to measure the industrial water use efficiency in the second stage of the four-stage DEA model. We placed the decision-making units (31 provinces in China) in the same external environment so that the water use efficiency of each province could be measured in the same external environment. We considered the external variables of different provinces to be the macroeconomic policies and structural factors of industry. Based on the literature on the impact of industrial water use efficiency, four external variables were selected: (1) the amount of investment in industrial pollution control (government influence), (2) the rate of urbanization (urbanization level of each province), (3) the proportion of the output value of state-owned industrial enterprises to the total industrial output value (institutional structure), and (4) the proportion of industrial added value to the GDP (industrial structure).

(4) Data sources

According to the above information, the indicators of each part are summarized in Table 1.

**Table 1:** Indicators of the four-stage DEA.

	Factor	Evaluation indicator
Input	Labor	Total number of industrial employees
	Capital	Industrial total assets
	Water	Industrial water consumption
	Pollutant	COD
		NH_3_
Output	Total industrial output value	Industrial sales value
External variables	Government influence	Amount of investment in industrial pollution control
	Urbanization level of each province	Rate of urbanization
	Institutional structure	Proportion of the output value of state-owned industrial enterprises to the total industrial output value
	Industrial structure	Proportion of industrial added value to GDP

This paper used the panel data of China’s 31 provinces during 2007–2016, Data sources can be found in the **Data Availability** section and the **S3 File**.

### 2.3 Data envelopment analysis considering the external environment

The four-stage DEA model is an evaluation method that can well examine the impact of exogenous environmental variables on efficiency. The basic idea of the four-stage DEA model is as follows.

The first stage: DEA-BCC model. This paper uses an input-oriented BCC model for analysis. The BCC model is used to determine the effectiveness of decision units under the assumption of variable returns to scale (VRS). The input-oriented BCC model can be expressed as follows:

$$\begin{array}{c} min\\ \theta ,\lambda \end{array}[\theta -X(s^{-}+s^{+})]$$

$$s.t.\;\sum_{i=1}^{n}\lambda_{i}y_{ir}-s^{+}=y_{0r}$$

$$\sum_{i=1}^{n}\lambda_{i}x_{ij}+s^{-}=\theta_{x_{0j}}$$ (1)

$$\sum_{i=1}^{n}\lambda_{i}=1$$

$$\lambda_{i}\geq 0,s^{+}\geq 0,s^{-}\geq 0$$

Where *i* = 1,2,…,n; *j* = 1,2,…,m; *r* = 1,2,…,s. *x*_*ij*_(*j* = 1,2,…,*m*) is the input elements; *y*_*ir*_(*r* = 1,2,…,*s*) is the output element; *θ* is the efficiency of the decision unit; *λ* is the weight; *s*^−^ and *s*^+^ are the output slack and input slack, respectively; and X is an infinitely small number of arbitrary positive numbers. The BCC model calculates an integrated technical efficiency, which can be decomposed into the product of scale efficiency and pure technical efficiency.

The DEA-BCC model can measure the technical efficiency, pure technical efficiency and scale efficiency of each decision-making unit. Technical efficiency refers to the maximum output for a given input; scale efficiency refers to the performance degree of the scale economy; and pure technical efficiency refers to the technical and management efficiency, that is, the technical efficiency after removing the scale efficiency. The technical efficiency is equal to the scale efficiency multiplied by the pure technical efficiency.

The first phase of the DEA model cannot separate the influence of external environmental factors (uncontrollable factors) on efficiency, so the following analysis is needed.

The second stage: calculation of the of the decision-making unit, which are the input slack variables in each province. The value of the slack variables is the difference between the actual input in each region and the input of the most efficient scenario. Since the input slack variables are at a minimum of zero, a data cut-off problem occurs. Therefore, the Tobit model is used for the analysis in this paper. We built m Tobit regression models (where m is the number of inputs). The explained variables are the total slack variables for each input (sum of ray slack variables and nonlaser slack variables), and the explanatory variables are the p observable exogenous environmental variables of the decision unit. The Tobit analysis of the input slack variables for each decision unit can be used to construct the following regression equation:

$$S_{ik}^{*}=f^{i}(z_{k},U_{i})+u_{ik}$$

$$S_{ik}=\left\{ \begin{array}{c} S_{ik}^{*},\;S_{ik}^{*}>0\\ 0,\;S_{ik}^{*}\leq 0 \end{array}\right.$$ (2)

Where *i* = 1,2,…,m; *k* = 1,2,…,n. *S*_*ik*_ = (1−*θ*_*k*_)*x*_*ik*_+*s*^−^ is the total value of the slack variables of the ith input calculated by the DEA-BCC model; *S*_*ik*_ is the ith input slack variable of the kth decision unit; *z*_*k*_ = (*z*_1*k*_,*z*_2*k*_,…,*z*_*pk*_) represents p observable environmental variables; *U*_*i*_ is the estimated parameter of the environmental variable; *f*^*i*^(*z*_*k*_,*U*_*i*_) represents the impact of the environmental variable *S*_*ik*_, where *f*^*i*^(*z*_*k*_,*U*_*i*_) = *z*_*k*_*U*_*i*_; and *u*_*ik*_ is a random error term.

The third stage: we use the Tobit regression model results to adjust the input of the decision unit further. The input of those decision-making units with a better environment was increased to eliminate the impact of external environmental. We adjusted the input of the other samples as follows:

$$\hat{x_{ik}}=x_{ik}+[max\{z_{k}U_{i}\}-z_{k}U_{i}]$$ (3)

Where *i* = 1,2,…,m; *k* = 1,2,…,n. *x*_*ik*_ represents the actual value of the i-th item of the k-th decision unit, $\hat{x_{ik}}$ is its adjusted value, and *U*_*i*_ is the estimated value of the environmental variable parameter. Formula (3) indicates that all decision units are adjusted to the same environment. The basic idea is that the maximum fitting slack value is equivalent to the worst external environment. When a decision unit is in the worst external environment, *max*{*z*_*k*_*U*_*i*_}−*z*_*k*_*U*_*i*_ = 0; then, the adjusted input $\hat{x_{ik}}=x_{ik}$, which is equivalent to having no adjustments to the initial investment. When a decision unit is under a good external environment, the adjusted input *max*{*z*_*k*_*U*_*i*_}−*z*_*k*_*U*_*i*_>0, which is equivalent to an improvement in the initial investment level. With the same output, the improvement of the input level will lead to a lower DEA efficiency score. Therefore, after the above adjustment process, all decision units can be adjusted to the same environment.

The fourth stage: The adjusted input data $\hat{x_{ik}}$ was obtained in the third stage replacement of the original input data *x*_*ik*_, with the output as the original output data *y*_*ik*_. The BCC model was used again for efficiency evaluation, and the resulting efficiency of each decision unit was the efficiency $\hat{\theta }$ after the external environmental factors were eliminated.

### 2.4 Factors influencing industrial water use efficiency

Industrial water use efficiency varies greatly among the 31 provinces, and many factors may affect the water use efficiency of each province. Because in the second stage of the four-stage DEA, the external variables were adjusted for the macroeconomic policies, the previously adjusted factors were not used in this section. Following the relevant literature[36,37,38,39,40], we divide the influencing factors into four aspects:

(1) Factor endowment aspect: we choose the per capita water resources to represent the water resource factor endowment of each province. This was calculated by dividing the total water resources of each province by the total population of each province.

(2) Economic aspect: ① Economic development level. We chose per capita GDP to represent the economic development level[40]. ② Scientific and technological progress. We choose funds for research and experimental development of industrial enterprises above a designated amount to represent scientific and technological progress. ③ Industrial structure. We chose the ratio of the output of eight industries with high water consumption to the total industrial output value to represent the industrial structures. China's water-intensive industries are concentrated in the power generating, textile, petroleum, pharmaceutical, food processing, paper-making, chemical fiber manufacturing and nonferrous metal smelting industries. These eight sectors account for 70% of the total industrial water use. ④ Proportion of foreign investment. As the driving force of economic development, foreign direct investment has formed two hypotheses regarding environmental effects: the pollution halo hypothesis[41] and pollution haven hypothesis[42]. The pollution halo hypothesis emphasizes that the advanced technologies created by foreign direct investment can improve environmental protection in developing countries. The pollution haven hypothesis maintains that enterprises in developed countries transfer high-pollution enterprises to developing countries with looser environmental regulations to avoid higher pollution control costs. To study the effect of foreign direct investment on China's environmental pollution, we considered the ratio of the total assets of foreign capital to the total assets of Chinese enterprises as an indicator to study the role of foreign direct investment in China's environmental pollution.

(3) Water resource utilization aspect. ① Water consumption per 10000 yuan of value-added by industry. ② Utilization rate of water resource development. We choose the ratio of total water consumption to total water resources to represent the utilization rate of water resource development. ③ Annual growth rate of industrial water use. ④ Industrial water price. The industrial water price has substantial influence on the utilization and allocation of water resources. In theory, the price can regulate industrial water consumption. Industrial water demand and industrial water price are inversely proportional. However, each province set different water prices for different industries and even different enterprises. The calculation method for the industrial water price varies from province to province. In addition, it is very difficult to collect data on industrial water prices. Therefore, we did not conduct a quantitative analysis of the industrial water price.

(4) Environmental education and governance. ① Industrial sewage treatment capacity. Sewage treatment capacity not only has a direct influence on pollutant discharge, but is also a reflection of technical level. We considered the facilities for the treatment of waste-water capacity as the indicator. (2) Educational investment. Generally, public awareness of environmental protection will increase with the improvement of education levels. We considered the per capita education expenditure as the indicator.

From the above, the following ten factors affecting industrial water use efficiency were selected for quantitative analysis, as is shown in Table 2. Data sources can be found in the **Data Availability** section and the **S4 File**.

**Table 2:** Indicators of factors affecting water use efficiency.

Aspect	Factor	Evaluation indicator
Factor endowment	Factor endowment	Per capita water resources
Economic	Economic development level	Per capita GDP
	Scientific and technological progress	Funds for research and experimental development of industrial enterprises above a designated amount
	Industrial structure	Ratio of the output value of eight major water-consuming industries to the total industrial output value
	Proportion of foreign investment	Ratio of the total assets of foreign capital to the total assets of Chinese enterprises
Water resource utilization	Water consumption per 10000 yuan of value-added by industry	Water consumption per 10000 yuan of value-added by industry
	Utilization rate of water resources development	Ratio of the total water consumption to the total water resources
	Annual growth rate of industrial water use	Annual growth rate of industrial water use
Environmental education and governance	Industrial sewage treatment capacity	Facilities for treatment of capacity of waste water
	Educational investment	Per capita education expenditure

## 3 Results and discussion

### 3.1 Industrial water use efficiency measurement without considering pollutant discharge

This section describes the four-stage DEA used to calculate the industrial water use efficiency without considering the pollutant discharge of Chinese provinces from 2007 to 2016, including the technical efficiency, pure technical efficiency and scale efficiency. The final calculation results are shown in the S1 File.

(1) Analysis of interannual variation of each efficiency value.

**Fig 2:**
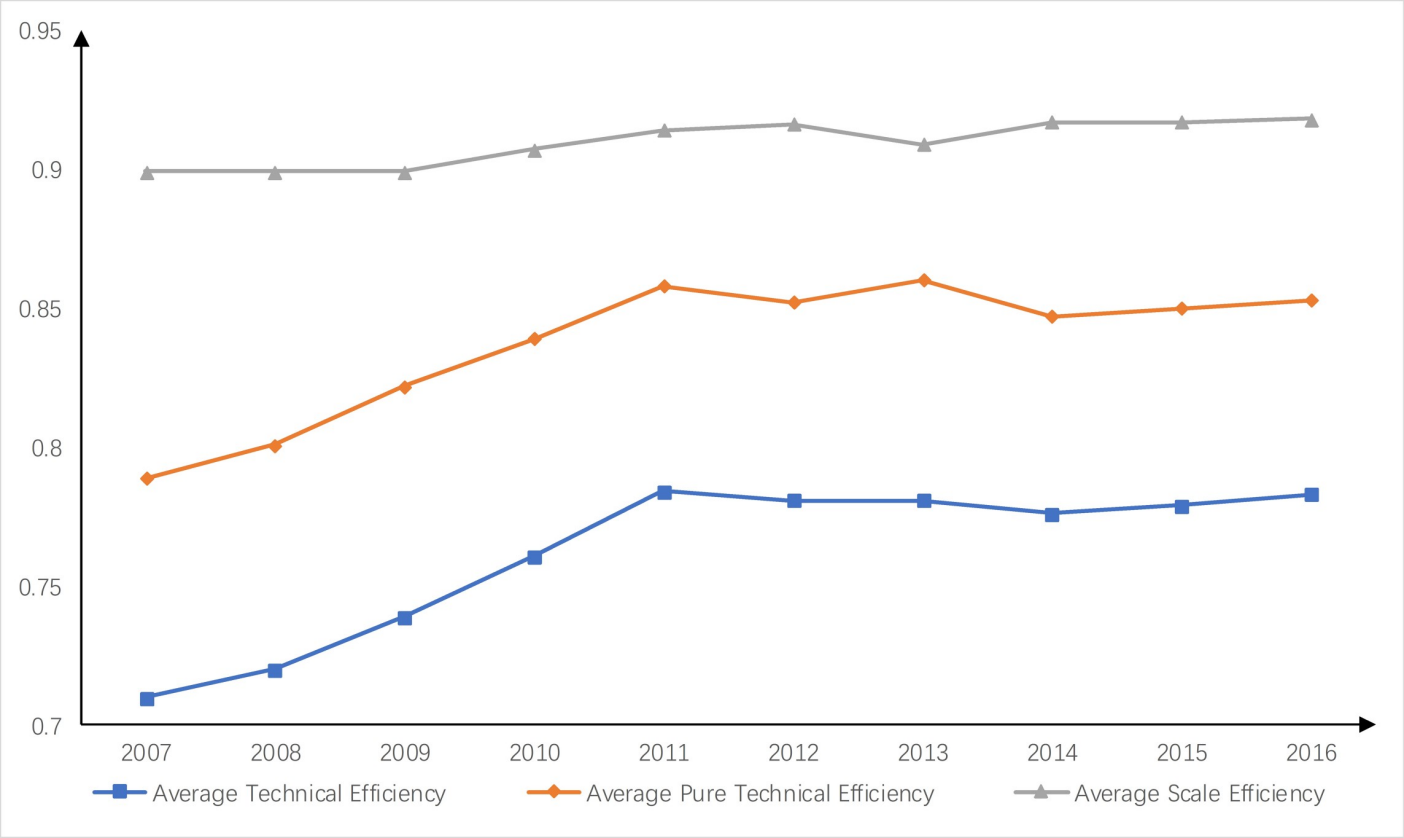
Comparison of average water use efficiency from 2007 to 2016.

As shown in Fig 2, the average scale efficiency of the industrial water resource use in China's 31 provinces was high from 2007 to 2016, while both the average technical efficiency and the average pure technical efficiency increased initially and then decreased. The average technical efficiency increased each year from 0.71 in 2007 to 0.784 in 2011, and then fluctuated to 0.783 in 2016.

The scale efficiency of the industrial water use was relatively high, but the pure technical efficiency was relatively low. We suggest that the government should strengthen the support for industrial water-saving policies, water-saving technologies and the update of water-saving equipment and increase the reuse rate of industrial water. These measures can improve the pure technical efficiency.

(2) Analysis of industrial water use efficiency in each province

**Fig 3:**
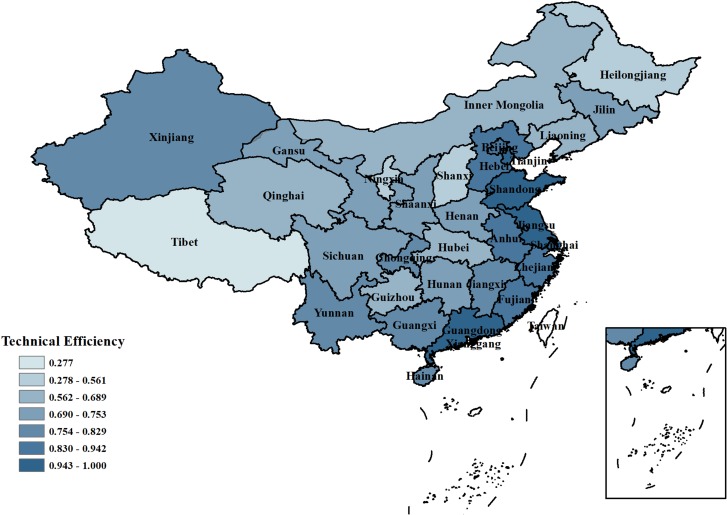
Comparison of average technical efficiency of industrial water resource use in 31 provinces of China.

**Technical efficiency:** As shown in Fig 3, the eastern coastal provinces have higher industrial water use technical efficiency. The industrial water efficiency of Tianjin, Shandong, and Guangdong reached 1, and the average technical industrial water use efficiency of Jiangsu, Zhejiang, and Beijing was close to 1. In central China, the industrial water use technical efficiency was approximately the same as the average in China. Most provinces in northeast and northwest China had a lower than average technical efficiency. Among them, the technical industrial water use efficiency of industrial water use in Tibet was the lowest, far lower than that of other provinces. Because the average elevation of Tibet is over 4000 meters, the implementation of water conservancy projects is complicated, and the progress of Tibet’s water conservancy projects is relatively lower than that of other provinces. Therefore, we suggest that the government should strengthen the adoption of water conservancy projects in Tibet.

**Fig 4:**
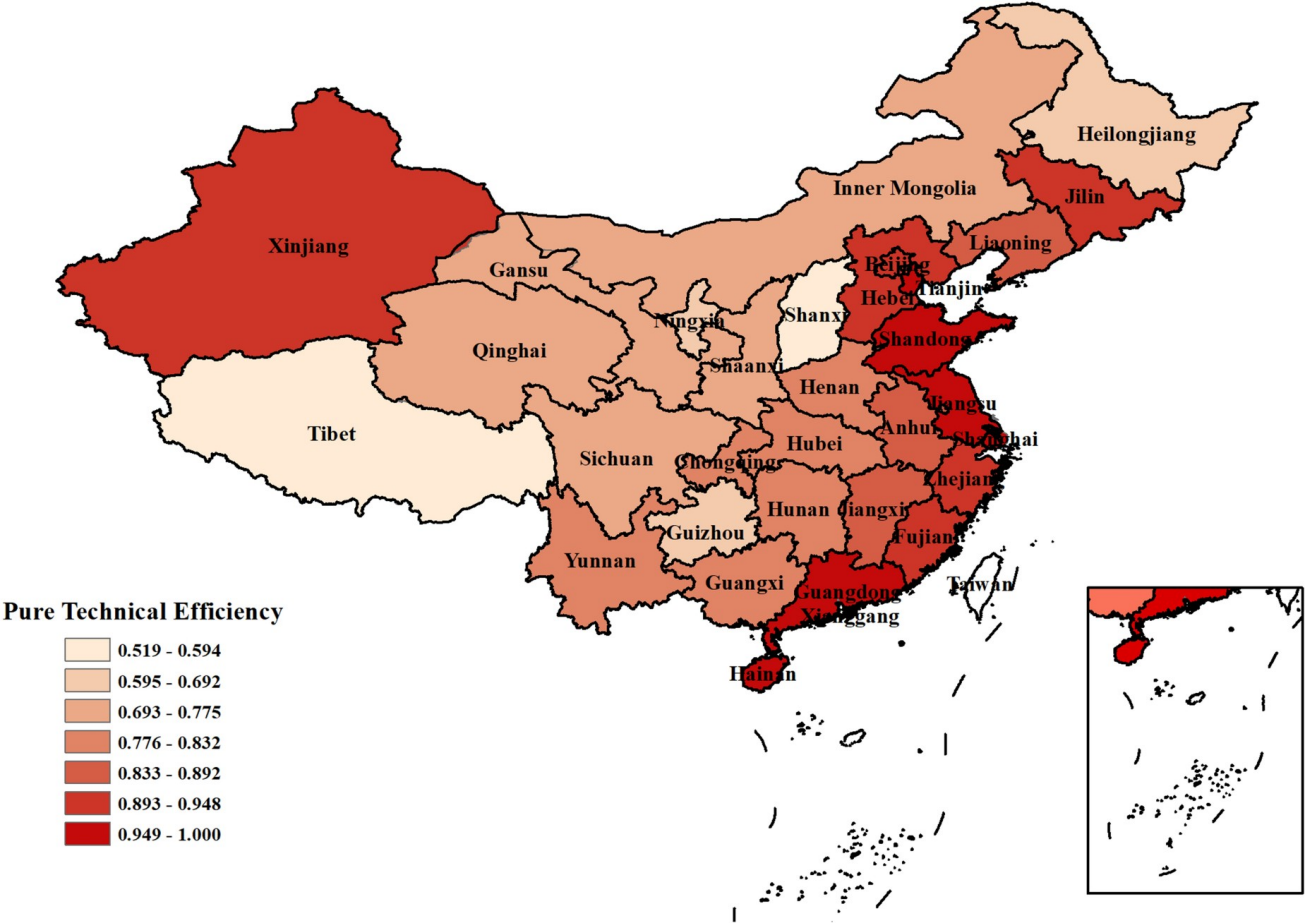
Comparison of the average pure technical efficiency of industrial water resource use in 31 provinces of China.

**Pure technical efficiency**: Industrial water use pure technical efficiency is the technical efficiency after removing the scale economic efficiency. As shown by the calculation results and Fig 4, the same spatial distribution law roughly applies to both the industrial water use pure technical efficiency and technical efficiency of China. The eastern coastal provinces still have the highest industrial water use pure technical efficiency, the pure technical efficiency of Jiangsu, Shandong, and Guangdong industrial water use reached 1, and that of Shanghai and Hainan was above 0.95. Compared with the national pure technical efficiency of industrial water use, the pure technical efficiency of industrial water use in the three northeastern provinces (Jilin, Liaoning and Heilongjiang) was greatly improved, which indicated that the water-saving technology of the three northeastern provinces helped to make the pure technical efficiencies of these areas better than the national average.

**Fig 5:**
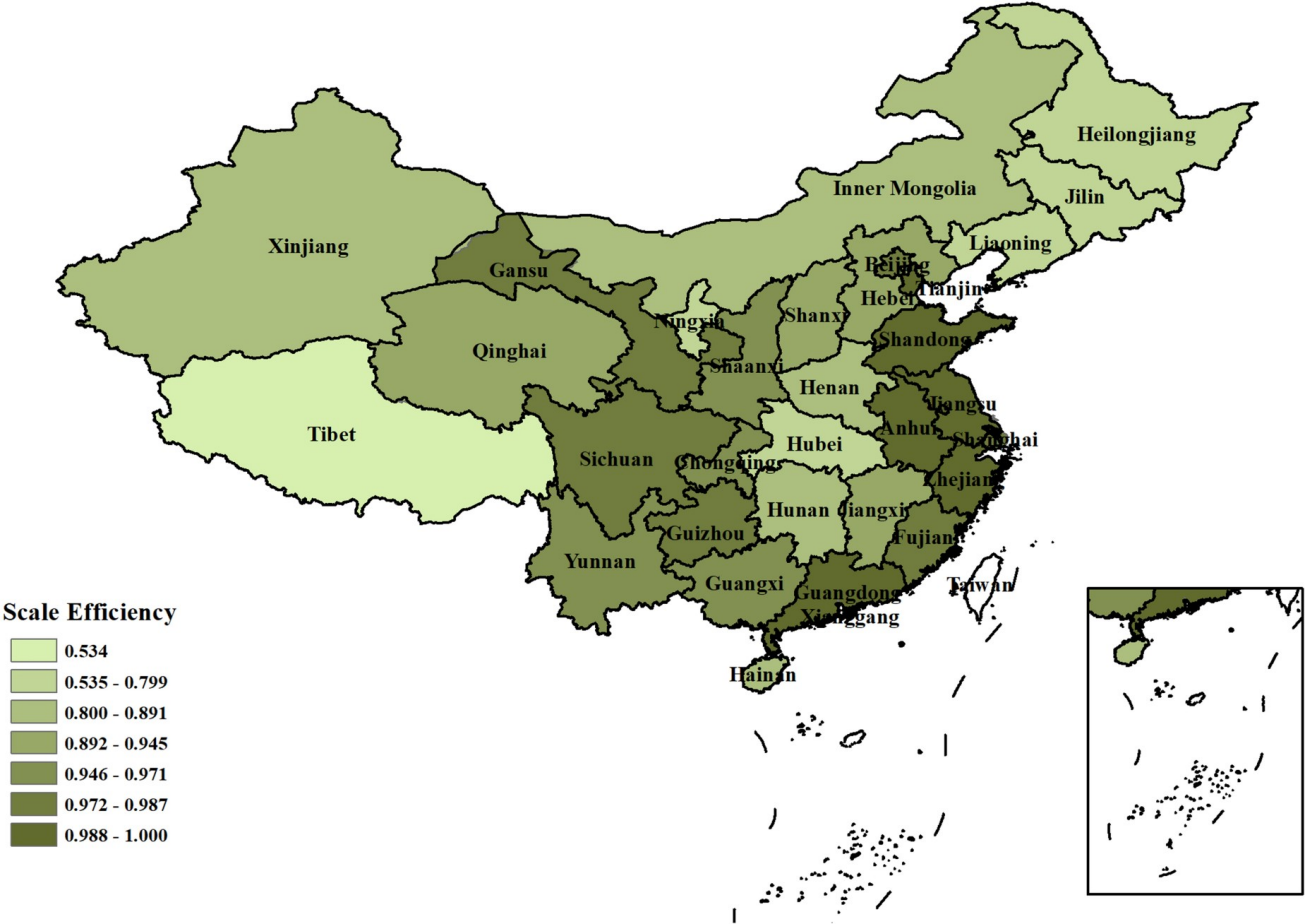
Comparison of average scale efficiency of industrial water resource use in 31 provinces of China.

**Scale efficiency:** The industrial water use scale efficiency in China is relatively high, with an average scale efficiency value of 0.91. The industrial scale efficiency in Tibet is relatively low, and the industrial water use efficiency is 0.534, far lower than the average level in China. As shown in Fig 5, industrial water use efficiencies in Jilin, Heilongjiang and Liaoning were higher than that in Tibet. The iron and steel industry in the three northeastern provinces consumes much water, and almost all production processes require much water. The steel industry accounts for 10% of the country's industrial water consumption, and new water consumption accounts for approximately 14% of the country's industrial water consumption. We suggest that the iron and steel industry in the three northeastern provinces should rely on technological progress, and adopt new water-saving technologies. These areas can improve the circulating water systems, expand the use of unconventional water sources, and reduce the consumption of water for new product.

### 3.2 Industrial water use efficiency measurement considering pollutant discharge

This section describes the four-stage DEA used to calculate the industrial water use efficiency considering the pollutant discharge of Chinese provinces from 2007 to 2016, including the technical efficiency, pure technical efficiency, and scale efficiency. The final calculation results are shown in the S2 File.

(1) Comparison of China’s industrial water uses technical efficiency with and without accounting for pollutant discharge.

**Fig 6:**
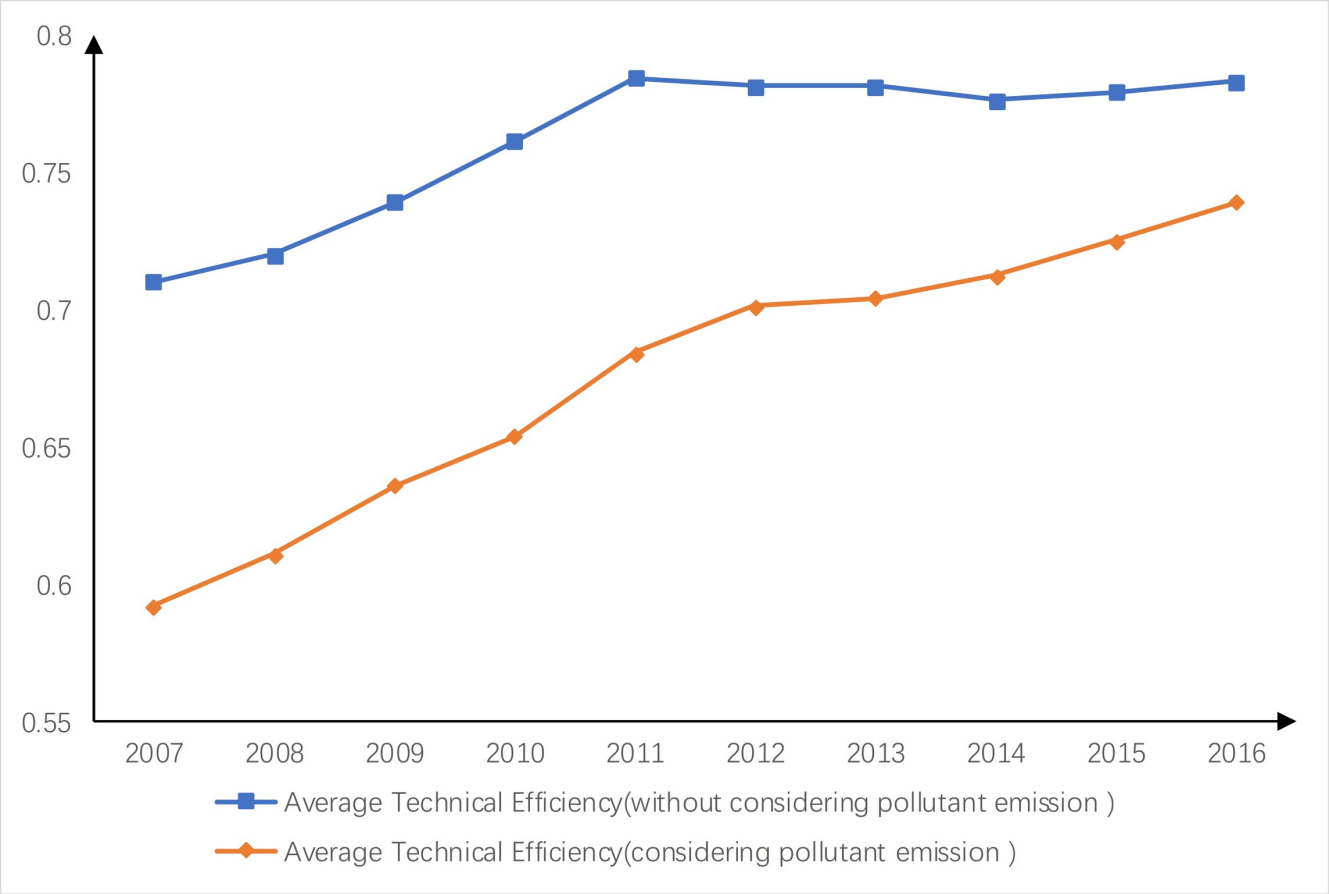
Comparison of average technical efficiency of industrial water resource use with and without accounting for pollutant discharge.

As shown in Fig 6, the average technical efficiency of industrial water use in China when pollutant discharge is considered has been on the rise, but it is still lower than the average technical efficiency of industrial water use in China when pollutant discharge is not considered. However, the gap between the two efficiencies has been narrowing over time, and the impact of pollutant discharge on the average technical efficiency of industrial water use has decreased. In recent years, China has continuously strengthened the regulation of industrial pollutant discharge, established adequate wastewater treatment plants, and reduced the cost of wastewater treatment, and qualified wastewater is now used for municipal green water. We recommend that the government continue to strengthen the relevant strategies to further reduce the impact of industrial pollutant on the average technical efficiency of industrial water use.

(2) Comparison of industrial water use technical efficiency in each province with and without taking pollutant discharge into account.

**Fig 7:**
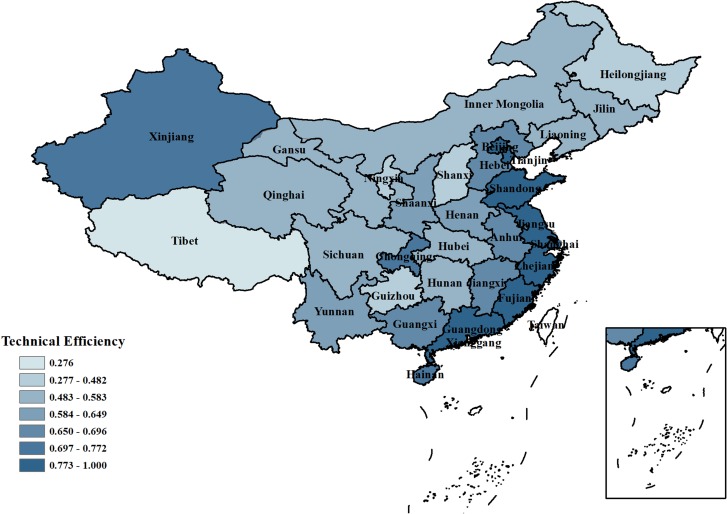
Comparison of average technical efficiency of industrial water resource use in 31 provinces of China when considering pollutant discharge.

As shown in Fig 7, the industrial water use efficiency considering the pollutant discharge of Zhejiang, Shanghai, and Tianjin was 1, while that of Beijing, Jiangsu, Shandong and Guangdong was close to 1. These provinces are all developed coastal provinces. With industrial upgrading, heavily polluting industries are gradually transferred to the central and western provinces, therefore, the industrial water use efficiency of these provinces is not affected by the pollutant discharge. Shanxi, Guizhou, Ningxia, Heilongjiang, and Tibet still have the lowest average industrial water use efficiencies in China.

**Fig 8:**
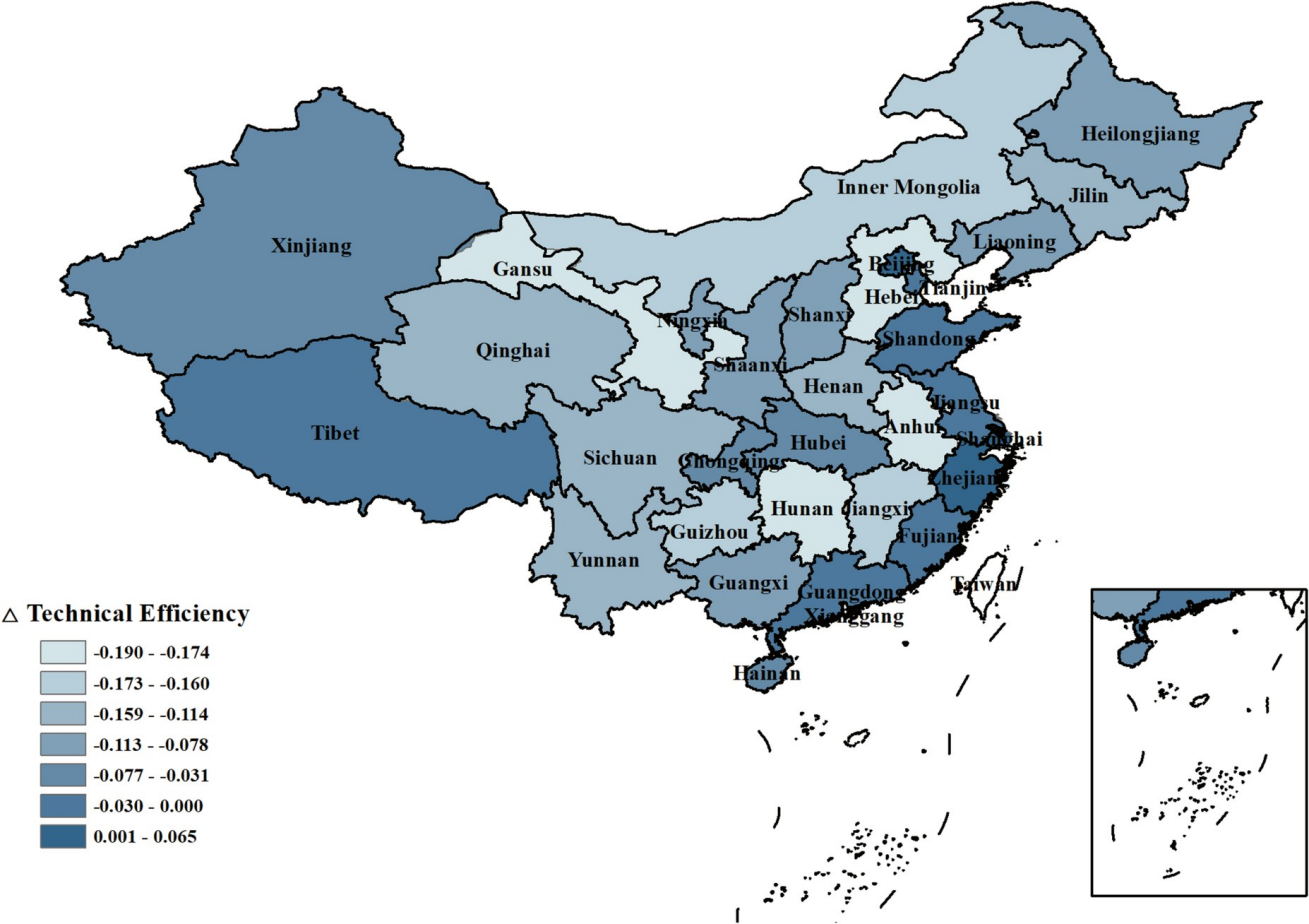
The difference between the average technical efficiency of industrial water resource use with or without accounting for pollutant discharge in 31 provinces of China.

Because carbon dioxide and ammonia nitrogen discharge are considered in the model, the industrial water use efficiency of each province changes. As shown in Fig 8, the technical efficiency of industrial water use increased when accounting for pollutant discharge in Beijing, Shanghai and Zhejiang. The water use efficiency of Tianjin was not affected by pollutant discharge and remained at 1. However, Anhui, Hebei, Hunan, Gansu, Guizhou, Jiangxi and other central provinces had the most significant decline in water use efficiency. These provinces have heavily polluting industries, and they are also undertaking the transfer of heavily polluted industries brought about by China's economic transformation.

In recent years, the water quality of developed coastal provinces has been relatively inferior. The water quality levels of the Yangtze River Estuary, Zhuhai Estuary, Hangzhou Bay and Taihu Lake have ranged between the fourth and fifth levels. This represents nearly the lowest level of the surface water quality standards in China. The local government has also strengthened the treatment of pollutants, therefore, the local heavily polluting enterprises face cost increases or maybe even be shut down. However, transferring these heavily polluting enterprises to the midwest of China, on the one hand, will not improve the overall efficiency of industrial water use technology in China; on the other hand, less developed central and western provinces do not have higher fiscal revenue than the developed eastern provinces, and pollutant discharge management in the central and western provinces is relatively less strict than that in eastern provinces, so the transfer of heavily polluting industries may aggravate the pollution situation in China.

In this paper, Δ technical efficiency is equal to the water use efficiency considering pollutant discharge minus the water use efficiency without considering pollutant discharge.

(3) Interannual analysis of industrial water use efficiency in typically polluted provinces

We selected several provinces for which pollutant discharge had the most significant impact on the industrial water use efficiency. The selection criteria were the provinces where the difference between the industrial water use efficiency without considering pollutant discharge and the water use efficiency considering pollutant discharge is greater than 0.15; These provinces include Anhui, Hebei, Hunan, Gansu, Guizhou, Jiangxi, and Inner Mongolia.

**Fig 9:**
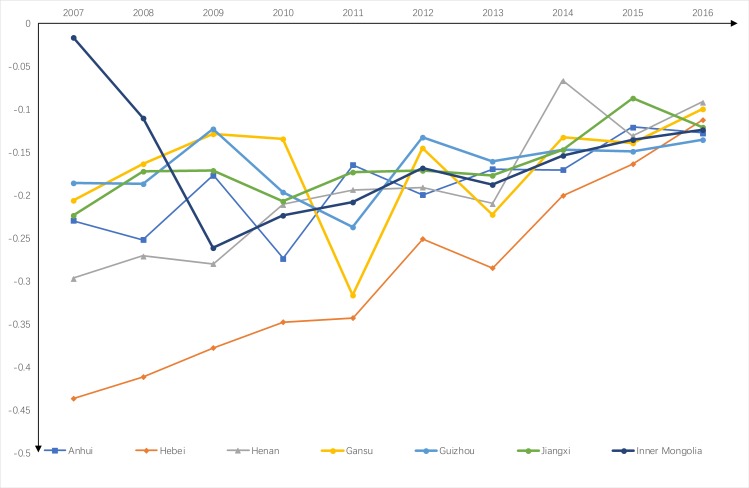
Interannual comparison of water use technical efficiency changes in heavily polluted provinces.

As shown in Fig 9, there was a significant difference between the water use technical efficiencies of heavily polluted provinces (except Inner Mongolia) with and without pollutant discharge in 2007. The difference in the technical efficiency of industrial water use in Hebei reached 0.437. However, the smaller difference in industrial water use efficiency in Inner Mongolia was due to the low water use efficiency when pollutant discharge was not considered. In the following years, the difference in the industrial water use efficiency in each heavily polluted province tended to decrease.

From the perspective of the whole country, the central and western provinces of China have maintained rapid economic growth. As the proportion of the industrial economy increases yearly, the total discharge of pollutants also shows a growing trend. However, statistical data show that the pollutant discharge shows decreasing trend, which indicates that local governments attach substantial importance to environmental problems. Because the economic development in the central and western regions has been dominated by energy and raw material processing industries for a long time, the base level of pollution is relatively large, and the discharge intensity in most regions is higher than the national average. In terms of the proportion of GDP in China, the central and western economies lag behind the eastern regions, but their discharge intensity is significantly higher than those of these regions.

On the one hand, the number of western enterprises is small and the economy is underdeveloped. On the other hand, environmental governance in the western region still has a long way to go. Polluting enterprises are not only the leaders of the regional economy but also sources of pollution that cannot be ignored. The coordination of the relationship with these enterprises warrants the attention of the local government.

### 3.3 Quantitative analysis of factors influencing industrial water use efficiency

A Tobit regression model was established to study the main factors affecting industrial water use efficiency in different provinces. Industrial water use efficiency is based on the technical efficiency of industrial water use in each province considering pollutant discharge. The Tobit model was described in the second stage of the four-stage DEA, and its characteristics are suitable for truncated data regression analysis. The technical efficiency value is used as the explained variable, and it is the truncated data between 0 and 1. Therefore, the Tobit model is appropriate to establish the regression model.The model is established as follows:

$$Y_{ij}^{*}=\alpha_{0}+\alpha_{1}{X1}_{ij}+\alpha_{2}{X2}_{ij}+\cdots +\alpha_{2}{X10}_{ij}+u_{ij}$$ (4)

$$Y_{ij}=\left\{ \begin{array}{c} Y_{ij}^{*},\;0<Y_{ij}^{*}\leq 1\\ 0,\;Y_{ij}^{*}\leq 0 \end{array}\right.$$ (5)

Where *i* = 1,2,3,…,31; *j* = 2007,2008,…,2016, *Y*_*ij*_ refers to the technical efficiency value in the *i*th province in the *j*th year, the results are shown in Table 3.

**Table 3:** Regression results of factors influencing industrial water use efficiency.

Aspect	Factor	Technical efficiency (Y)
Factor endowment	Per capita water resource (X1)	-1.99*** (-2.87)
Economic	Economic development level (X2)	7.48*** (4.36)
	Scientific and technological progress (X3)	-1.62** (-2.16)
	Industrial structure (X4)	-1.22** (-1.77)
	Proportion of foreign investment (X5)	1.33** (0.84)
Water resource utilization	Water consumption per 10000 yuan of value-added by industry (X6)	1.93** (1.35)
	Utilization rate of water resources development (X7)	-0.17 (1.02)
	Annual growth rate of industrial water use (X8)	-0.65 (0.28)
Environmental education and governance	Industrial sewage treatment capacity(X9)	2.31** (1.25)
	Educational investment (X10)	3.54* (3.22)
Constant term		6.57** (4.62)
Wald chi2(10)		147.68***
Log likelihood		297.66

Note:*, **, *** represent significance at the level of 10%, 5%, and 1% respectively.

The test values are in parentheses.

As shown in Table 3, the Wald statistics of the model passed the significance test at the 1% level, which shows that the Tobit model with the technical efficiency value as the explained variable is significant. The following is a detailed analysis of each factor influencing the industrial water use efficiency.

(1) The coefficient of factor endowment was negative, it passed the significance test at the 1% level, and it showed that per capita water resources have a significant negative correlation with technical efficiency. When the per capita water resources are high, a reduction in industrial water prices occurs. To some extent, this reduces the motivation of industrial enterprises to save water, and cause low industrial water use efficiency.

(2) The coefficient of economic development level was positive, it passed the significance test at the 1% level and showed that per capita GDP has a significant positive correlation with technical efficiency. With the rapid development of the economy and the acceleration of industrialization, people are increasingly aware of the importance of saving water, and there is also enough tax revenue to improve water-saving technologies.

(3) The coefficient of scientific and technological progress was negative, it passed the significance test at the 5% level and showed that funds for research and experimental development of industrial enterprises above a designated amount were negatively correlated with technical efficiency. This is a result that contradicts theory and intuition. However, after careful analysis of the data, we find that China's industrial water use efficiency increased by less than 10% on average from 2007 to 2016. Meanwhile, China's funds for research and experimental development of industrial enterprises above a designated amount have risen by an average of more than 10 times. The funds for research and experimental development of industrial enterprises above a designated amount are relatively lower than the industrial water use efficiency. Therefore, the coefficient of science and technology is negative.

(4) The coefficient of the industrial structure is negative, it passed the significance test at the 5% level and showed that the ratio of the output of eight high-water-consuming industries to the total industrial output has a negative correlation with technical efficiency. These eight high-water consuming industries account for approximately 70% of the industrial water use, and mainly represent the extensive mode of production. The government should attach great importance to these water-consuming industries and take measures to improve the reuse rate of industrial water.

(5) The coefficient of proportion of foreign investment was positive, it passed the significance test at the 5% level, it shows that the ratio of the total assets of foreign capital to the total assets of Chinese enterprises has a significant positive correlation with technical efficiency. It supports the pollution halo hypothesis. Regarding the water environment in China, the Chinese government has a relatively strong supervision over foreign capital. According to the statistical results, enterprises invested by foreign capital do not pollute the environment.

(6) The coefficient of water consumption per 10000 yuan of value-added by industry was positive, it passed the significance test at the 10% level, it shows that water consumption per 10000 yuan of value-added by industry has a significant positive correlation with technical efficiency. Water consumption per 10000 yuan of value-added is an important indicator for China to implement the strictest water resources management. Through this indicator, the Chinese government can better urge industrial enterprises to carry out water-saving transformation.

(7) The coefficient of industrial sewage treatment capacity was positive, it passed the significance test at the 5% level, it shows that facilities for the treatment of waste water capacity have a significant positive correlation with technical efficiency. Industrial sewage treatment plants are important infrastructures of a city. In recent years, the industrial sewage treatment capacity of developed cities such as Beijing and Shanghai have been gradually declining due to industrial transfer, but whether the infrastructure of less developed cities can keep up with the pace of industrial transfer is a worrisome issue.

(8) The coefficient of educational investment was positive, it passed the significance test at the 10% level, it shows that per capita education expenditure have a positive correlation with technical efficiency. Because of the transfer payment from the central government, the investment in education is not directly proportional to the local economy, but there is still a positive correlation. Education takes many forms. Shanghai is implementing strict waste separation policies, which will undoubtedly make Shanghai citizens pay more attention to the environment. However, such policy spending does not count towards education spending. As part of future research, we will further decompose the indicators of educational investment to make it more scientific.

(9) The utilization rate of water resources development and the annual growth rate of industrial water use did not pass the significance test, indicating that these two factors have little influence on industrial water use efficiency in China.

## 4 Conclusion

We applied the four-stage DEA model to calculate the technical efficiency, pure technical efficiency and scale efficiency of industrial water resource use in 31 provinces from 2007 to 2016. We first analyzed the water use efficiency of the Chinese industry without considering pollutant discharge, and the results showed the following:

1. China's industrial water use efficiency increased from 0.71 in 2007 to 0.784 in 2011, and then it remained relatively stable. The scale efficiency of industrial water use in China is approximately 0.9, while the pure technical efficiency is relatively low.
2. The industrial water use efficiency of coastal provinces is higher than that of the central and western regions, among which the water use efficiencies of Tianjin, Shandong, Guangdong and other provinces are close to 1, while the water use efficiency of Tibet is far lower than those of other provinces.
3. The eastern coastal provinces still lead the country in pure technical efficiency. Compared with the interprovincial scale efficiency, the scale efficiency of Heilongjiang, Jilin and Liaoning is only higher than that of Tibet, which indicates that the water consumption by the iron and steel industry in the three northeastern provinces is still relatively extensive.

We then calculated the industrial water use efficiency considering pollutant discharge and compared it with the water use efficiency without considering pollutant discharge. The results showed that:

1. The national industrial water use efficiency decreased significantly after considering the pollutant discharge. However, the gap between the two efficiencies became decreased over time.
2. The industrial water use efficiency of the eastern coastal provinces is still the highest in China, and the water use efficiency is not affected by pollutant discharge. However, the water use efficiency of the central and western provinces such as Anhui, Hebei, Hunan, Gansu, Guizhou and Jiangxi has decreased the most.
  With industrial transfer in China, heavily polluting enterprises are gradually transferred from the eastern to the central and western regions, but the pollution control facilities in the central and western provinces have more shortcomings than those in the eastern provinces. If we do not pay attention to governance, the industrial transfer of heavily polluting enterprises may cause greater pollution to the overall environment of the country.
3. Affected by the pollutant discharge, the heavily polluted provinces (except Inner Mongolia) had the largest decline in the industrial water use efficiency in 2007. Over time, the decline in industrial water use efficiency was gradually alleviated. Although heavily polluting enterprises have brought economic growth to the central and western provinces, balancing the environment and economy remains a challenge for the local government.

Finally, we analyzed the factors influencing industrial water use efficiency. According to the relevant literature, we selected ten elements for this analysis. Among them, factor endowment, economic development level, scientific and technological progress, industrial structure, proportion of foreign investment, water consumption per 10000 yuan of value-added by industry, industrial sewage treatment capacity and educational investment passed the significance test. There is a positive correlation between economic development level, proportion of foreign investment, industrial sewage treatment capacity, educational investment, water consumption per 10000 yuan of value-added by industry and industrial water use efficiency. Factor endowment, scientific and technological progress, industrial structure and industrial water use efficiency are negatively correlated. The utilization rate of water resources development and the annual growth rate of industrial water use did not pass the significance test.

## Supporting information

S1 File Comparison of industrial water use efficiency without considering pollutant discharge factors of 31 provinces in China from 2007 to 2016. (XLSX)

S2 File Comparison of industrial water use efficiency considering pollutant discharge factors of 31 provinces in China from 2007 to 2016. (XLSX)

S3 File Indicators of the four-stage DEA. (XLSX)

S4 File Factors influencing industrial water use efficiency. (XLSX)
